# IR and NMR Studies of the Status of Al and Acid Sites in Desilicated Zeolite Y

**DOI:** 10.3390/molecules25010031

**Published:** 2019-12-20

**Authors:** Mariusz Gackowski, Jerzy Podobiński, Ewa Broclawik, Jerzy Datka

**Affiliations:** Jerzy Haber Institute of Catalysis and Surface Chemistry, Polish Academy of Sciences, Niezapominajek 8, PL-30239 Krakow, Poland; ncgackow@cyf-kr.edu.pl (M.G.); ncpodobi@cyf-kr.edu.pl (J.P.); datka@chemia.uj.edu.pl (J.D.)

**Keywords:** zeolite Y, desilication, IR spectroscopy, NMR spectroscopy, EFAL forms, acid sites

## Abstract

The desilication of zeolite Y (of Si/Al = 31) that was previously dealuminated by steaming and acid treatment was studied. Desilication of zeolites of high Si/Al module in alkali solutions extracts both Si and Al from zeolite crystals, but while Si remains in solution, Al is reinserted into the zeolite grain. The main goal of our study was to follow the status of Al reinserted into zeolite during the desilication procedure, and its role in the formation of acid sites of the Brønsted and Lewis types. The properties of Al were followed by ^27^Al MAS NMR spectroscopy (for parent samples and zeolites treated either with NaOH or NaOH/tetrabutylammonium hydroxide), whereas the acid sites generated in the final stages were studied by IR spectroscopy with NH_3_ and CO as probe molecules. In non-desilicated zeolite, most of the Al was in a typically zeolitic tetrahedral coordination, while both NMR and quantitative IR studies of NH_3_ sorption evidenced that Al that was extracted by desilication and was subsequently reinserted had a tetrahedral coordination similar to amorphous aluminosilicates and showed an ion exchange ability. After the exchange of Na^+^ to NH_4_^+^ and decomposition of NH_4_^+^ ions, reinserted Al forms generated protonic sites from which some condensed at higher temperatures producing Lewis acid sites (with stoichiometry typical for zeolites i.e., the condensation of two protonic sites produces one Lewis site) but some other kept their character.

## 1. Introduction

Zeolites are very important catalysts in the chemical industry, especially in oil refineries. The most important advantages of zeolites as catalysts are related to the fact, that active sites are situated inside the micropores and the carbocations produced by the addition of protons to reactants are stabilized by the negative charge of the framework. Moreover, shape selectivity occurs in some zeolites. Another advantage of zeolites is the presence of very strong Brønsted acid sites. However, the disadvantage of zeolites might be a restricted diffusion of reactants in micropores. One method of improvement of the catalytic efficiency of zeolites is the preparation of hierarchical zeolites with developed pore system comprising mesopores. The desilication of zeolites in alkaline solutions turned out to be the most effective way of producing mesoporous zeolites. A lot of data on the synthesis of mesoporous zeolites, their properties, and catalytic applications were presented in a monograph edited by J. Garcia-Martinez and Kunhao Li [1]. Discussion concerning the mechanism of desilication has been presented in [2,3,4,5,6]. Even though a lot of desilication studies have been realized using various zeolites, the majority of these studies were focused on ZSM-5 type zeolite [7,8,9,10,11,12,13,14,15,16,17], the composition of which (Si/Al = 30–50) is optimal for desilication so the samples of good porosity, acidity, and catalytic activity could be prepared. Desilication of Y zeolites has been much less frequently studied. A standard zeolite Y with Si/Al ratio around 2.5 contains numerous AlO_4_^−^ groupings protecting the zeolite framework against the OH^−^ attack. On the other hand, a standard zeolite Y subjected first to dealumination by steaming followed by acid leaching to yield a high-silica material with Si/Al > 15 is amenable to the reaction with OH^−^ groups. Such zeolites were, however, unstable and could be destroyed not only in a diluted NaOH solution [18], but also in highly diluted ammonia solutions [19,20]. The addition of tetrapropylammonium ions (TPA^+^) to NaOH turned to be a crucial modification of the desilication route since TPA^+^ well protects the zeolite structure upon mesopores formation [21,22,23,24].

We have already studied the desilication of zeolite Y of Si/Al = 31 (FAU-31) with NaOH/tetrabutylamine hydroxide (TBAOH) mixture [25,26,27] and zeolites of high acid strength of Si-OH-Al groups, and obtained a good porosity and good catalytic properties. A very interesting observation was that in some cases, the Si-OH-Al groups of extremely high acidity (Δν_OH...CO_ = 410 cm^−1^) were formed during the calcination of zeolite containing TBA^+^ ions in atmospheric air [26].

The present study details the status of Al in FAU-31 zeolite (parent and desilicated with either NaOH or NaOH/TBAOH mixture) and the formation of acid sites in these zeolites under further treatment. In agreement with former suggestions based on mechanistic consideration of desilication and dealumination [2], both Si and Al are extracted from zeolite crystals under alkaline treatment, but while Si remains in the solution, Al is reinserted back into the zeolite. The precise goal of our study was to follow the status of such reinserted Al and its role in the formation of acid sites of Brønsted and Lewis types. The properties of Al were followed by ^27^Al MAS NMR spectroscopy and the acid sites were studied by IR spectroscopy with NH_3_ and CO as probe molecules.

## 2. Results and Discussion

**Figure 1:**
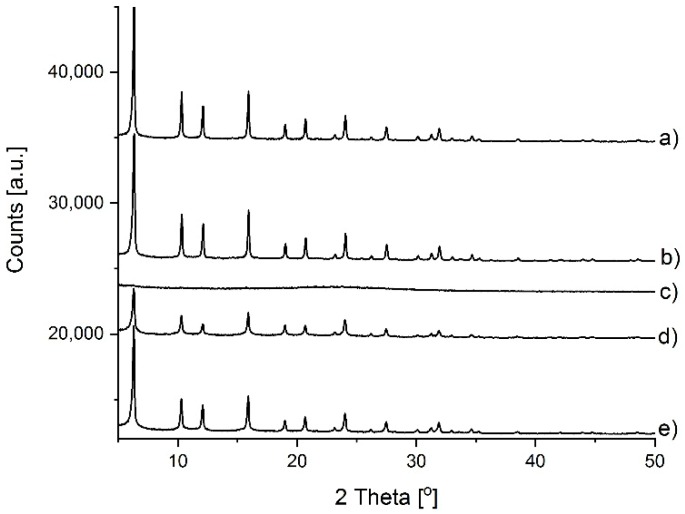
XRD diffractograms of the samples: (**a**) FAU-31, (**b**) FAU-31 calc., (**c**) NaOH; NH_4_^+^, (**d**) NaOH/TBAOH; NH_4_^+^, and (**e**) NaOH/TBAOH; NH_4_^+^ calc.

Before further characterization, an XRD analysis was performed (Figure 1) to check to what extent the crystal structure of the sample was retained after the treatment with desilicating agents. The results of the chemical analysis of zeolites and filtrates, as well as of the porosimetric studies are presented in Table 1. The treatment of zeolite with NaOH caused loss of ca. 79% of Si and of much smaller amounts of Al. The zeolite was completely amorphized, most of the microporosity was lost, and an amorphous material of relatively big mesoporosity was obtained. The NaOH/TBAOH mixture extracted smaller amount of Si, preserved most of the microporosity and produced mesopores of even bigger volume than NaOH alone.

**Figure 2:**
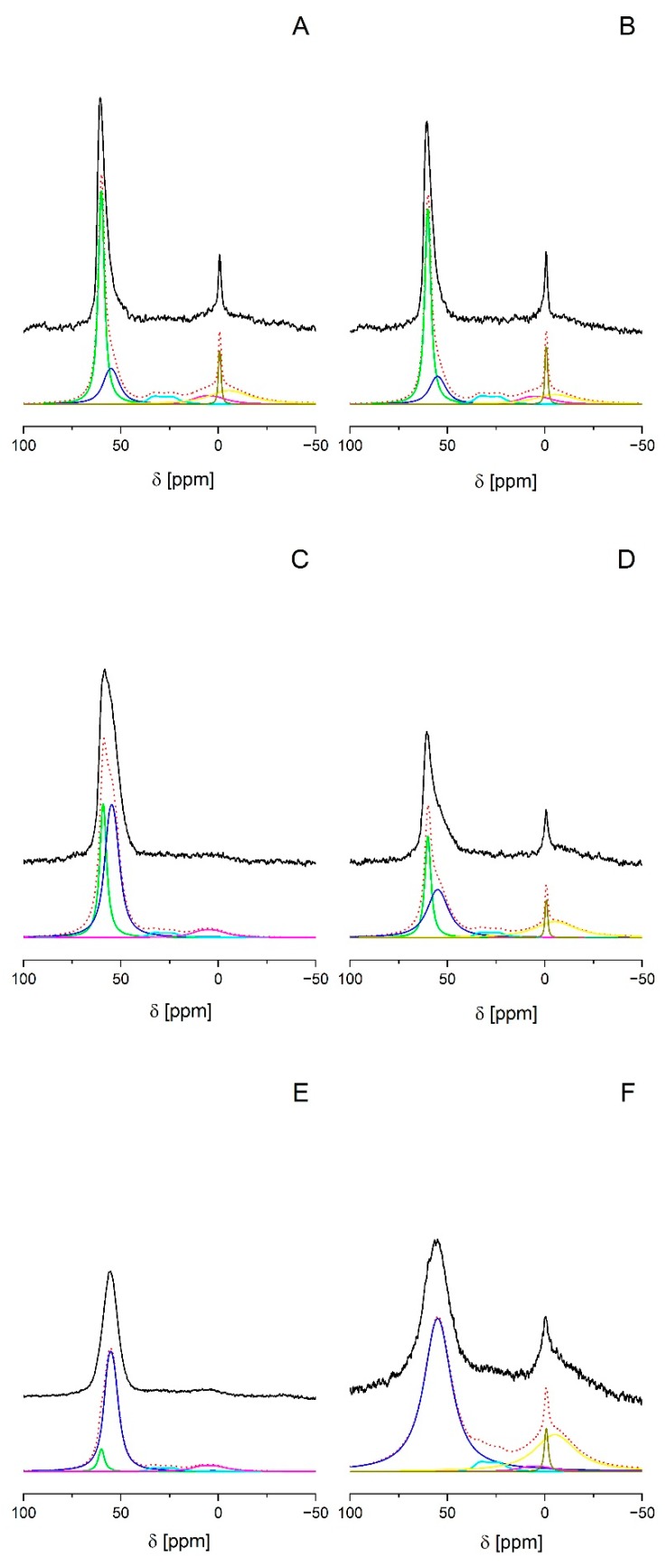
Deconvolution of ^27^Al MAS NMR spectra of the parent material FAU-31 (**A**,**B**), NaOH; NH_4_^+^ (**C**,**D**), NaOH/TBAOH; NH_4_^+^ (**E**,**F**); (**A**,**C**,**E**) denote samples before calcination and (**B**,**D**,**F**) denote the samples after calcination. Black—experimental spectrum, dotted—calculated spectrum, green—Al_IVa_, dark blue—Al_IVb_, cyan—Al_V_, pink—Al_VIa_, yellow—Al_VIb_, and olive—Al_VIc_.

**Figure 3:**
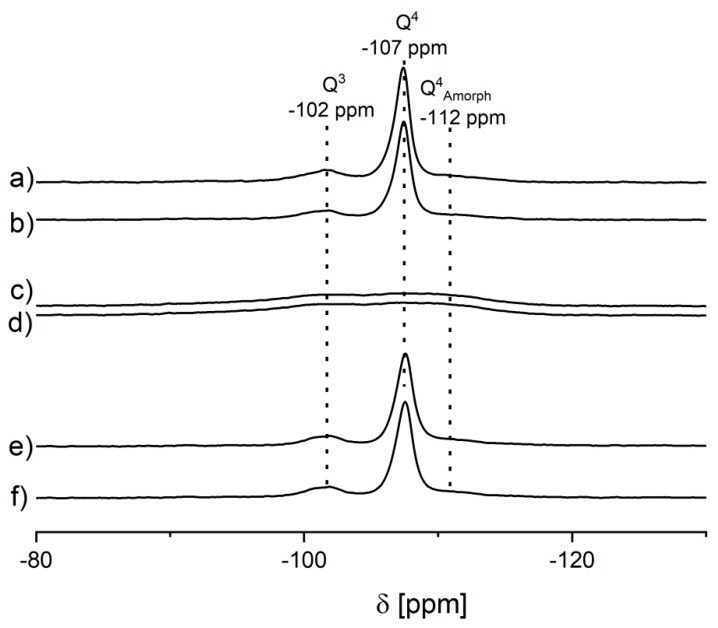
^29^Si MAS NMR spectra of the samples: (**a**) FAU-31, (**b**) FAU-31 calc., (**c**) NaOH; NH_4_^+^, (**d**) NaOH; NH_4_^+^ calc., (**e**) NaOH/TBAOH; NH_4_^+^, and (**f**) NaOH/TBAOH; NH_4_^+^ calc.

The ^27^Al MAS NMR spectrum recorded upon hydration of the parent sample, i.e., FAU-31 zeolite dealuminated by steaming and acid treatment (Figure 2A) shows several Al signals (Table 2, Figure 2A). The most important signal of δ_iso_ = 61 ppm and of quadrupolar constant (CQ) = 1.5 MHz (denoted as Al_IVa_), which is typical of tetra-coordinated zeolitic Al. The next close-lying signal of δ_iso_ = 59 ppm and of CQ = 3.2 MHz (denoted as Al_IVb_) might also be separated in the deconvoluted spectrum (Figure 2A). This signal exhibits high quadrupolar broadening and has been ascribed to tetrahedral Al distorted by highly charged extra-framework Al [28]. We supposed that such non-zeolitic Al might have already formed during the dealumination procedure. It was also considered that in the case of low Al content, extra-framework forms might stay close to a zeolite surface and affect the signal of near-surface Al yielding Al_IVb_. Another possibility was that it was connected with the amorphous phase generated during dealumination and desilication. Van Aelst et al. [19] observed that the intensity of Al_IVb_ signal correlated well with a decrease of crystallinity of the USY sample desilicated using NH_4_OH. By comparing the XRD data in Figure 1 with the results of deconvolution from Table 2, we can see that this trend was observed in our case as well. Some information could be also brought by the ^29^Si MAS NMR spectra (Figure 3). Besides the standard Q^4^ and Q^3^ signals at −107 ppm and −102 ppm, respectively, a broad signal at −112 ppm could be noticed. It was assigned to silica in amorphous phase (Q^4^_amorph_) [19]. ^27^Al spectra are much richer—apart from signals of tetrahedral form, deconvoluted spectrum showed four additional Al signals. One of them was assigned to penta-coordinated Al_V_ (δ_iso_ = 39 ppm and CQ = 5.5 MHz) and the other three signals were assigned to hexa-coordinated Al_VIa_, Al_VIb_, and Al_VIc_ of δ_iso_ = 14, 0.4, and 0 ppm and of CQ = 5, 4, and 1 MHz, respectively. High-coordinated forms of Al species could also be due to the coordination of additional water molecules upon hydration, as proposed by Zhao et al. [29] and by Li et al. [30].

Consecutive transformations of the sample (treatment by NaOH or NaOH/TBAOH followed by cation exchange, calcination, saturation with ammonia, and ammonium ions decomposition) changed the status of Al. Following the evolution of these Al forms by both NMR and IR might help to understand the mechanism of mesopore formation by alkaline desilication and of the formation of specific acid sites in the next steps.

Desilication of a zeolite with NaOH or NaOH/TBAOH resulted in, both, a decrease of the Si/Al ratio from 31 to 11 (NaOH) or to 17 (NaOH/TBAOH), and an increase of the Al content. The integrated Al signal in NMR spectrum increased accordingly (Table 2) but this increase (2.8 and 4.2 times for NaOH and NaOH/TBAOH, respectively) was ca. 1.5 times bigger than that predicted from Si/Al ratio given by the chemical analysis. This difference might be explained assuming that the parent sample (FAU-31) contained a significant amount of Al distorted from tetrahedral geometry, which experienced high quadrupolar coupling, making its signal so broad, it was basically ‘invisible’ to ^27^Al MAS NMR. We could assume that in ion-exchanged samples (so after desilication), this type of aluminum is not present [31]. This could lead to major discrepancies in quantitative examination of the samples under study.

^27^Al MAS NMR spectra also showed that the desilication (both in NaOH and NaOH/TBAOH) followed by the exchange of Na^+^ to NH_4_^+^ distinctly changed the distribution of Al among various sites (Figure 2C,E and Table 2). The contribution of Al_IVa_ decreased and the contribution of Al_IVb_ increased significantly. These two effects were more distinct after the treatment of a zeolite with NaOH. Two of the signals assigned to hexa-coordinated (octahedral) Al (Al_VIb_ and Al_VIc_, of δ_iso_ about 0.4 and 0 ppm, accordingly) disappeared. One possible interpretations assumed the dissolution of such Al species, whereas another one assumed the change of coordination of these Al forms from octahedral to tetrahedral in the presence of extra-framework cations [28,31,32,33,34]. Interestingly enough, the position of the Al_VIa_ signal of the octahedral species remained unchanged by the desilication process, while the chemical shifts of both signals assigned to the tetrahedral Al_IVa_ and Al_IVb_ forms underwent a shift in the sample treated with NaOH/TBAOH, in the comparison to the parent sample. A similar shift was observed in the ^27^Al and ^29^Si MAS NMR spectra for the La-exchanged zeolite and ultrastable zeolite [28]. It was ascribed to the change of the average T–O–T angle upon interaction with the positively-charged cations. In our case, the phenomenon could be ascribed to the interaction with cations such as TBA^+^.

As mentioned above, the process of Si dissolving during mesopore formation is accompanied by removal of a typical zeolitic four-coordinated Al_IVa_. However, while the Si remained in the solution, Al was found to have reinserted into a zeolite. This might be related to the increase of the contribution of the Al_IVb_ signal observed in the NMR spectrum recorded after the NaOH/TBAOH treatment, however, the provenience of the Al_IVb_ species was not clear. The nature and status of the Al and Si species after desilication and the details of the mechanism of mesopore formation in an alkaline environment at molecular level have already been discussed by Zhai et al. [2]. The relative stabilities of putative forms in the solution or after re-adsorption on a zeolite surface were considered, therein, based on the calculated binding energies in model systems built of Si–Si, Al–Si, or Al–Al double-T (T = Si or Al) fragments in solution, compared to adsorption energies between Si and the surface defect center or between Al and the surface defect center. These calculation results showed that the adsorption energies of the Si species on the studied surfaces were lower than the binding energies between the respective Si fragments in solution, which suggested that the Si species were prone to self-aggregation in solution. On the contrary, the adsorption energies of the Al species on the defected surfaces were higher than the binding energies between the corresponding fragments in solution, which indicated that the Al species preferred to be adsorbed on the surfaces. As a consequence, Si was effectively dissolved whereas Al remained in a zeolitic grain. This explained the selective loss of Si in alkali solutions. The second important consequence was that the selective removal of Si was accompanied by the effective enrichment of surface in Al forms, which might be responsible for hindering excessive mesopore formation.

Many authors suggested that the Al forms that were reincorporated into a zeolite surface have fourfold coordination. In line with this, our NMR spectra showed a distinct increase of the signal of Al_IVb_ after desilication, which seemed to suggest that this species, loosely linked to the surface of a parent FAU-31, might make a sort of bond with the surface oxygen in desilicated sample. This allowed us to speculate that such four-coordinated species of Al should also gain some ion-exchange capacity and, in consequence, it should generate a kind of protonic acid site, upon transforming Na^+^ to NH_4_^+^ and ammonium ions decomposition. In order to verify this hypothesis, the concentration of ammonium ions was determined by IR spectroscopy and compared with the concentration of Al obtained from chemical analysis for the corresponding samples. The information on the presence and concentration of both Brønsted and Lewis acid sites was obtained in IR experiments of ammonia adsorption. The interaction of ammonia with protonic sites produced ammonium ions (1450 cm^−1^ band) and the interaction with Lewis sites produced complexes for which the 1620 cm^−1^ band was typical.

Zeolites desilicated with NaOH or NaOH/TBAOH and treated with NH_4_NO_3_ showed the band at 1450 cm^−1^ that is typical of NH_4_^+^, and its intensity helped determine the concentration of NH_4_^+^. The extinction coefficient of this band was fixed in a separate experiment with measured doses of ammonia sorbed at 400 K in zeolite HY of Si/Al = 2.5. In this experiment, a linear plot of 1450 cm^−1^ band intensity versus the concentration of sorbed ammonia was found, which allowed to fix the extinction coefficient to the slope of this line as equal to 0.130 cm/µmol.

Concentrations of Al determined by chemical analysis for zeolites desilicated with NaOH and with NaOH/TBAOH are listed in Table 3; as compared to the concentration of ammonium ions in the desilicated samples, these subsequently transformed into the ammonium form and then dehydrated at 370 K (but not yet calcined). It might be seen that for both desilicating agents the concentrations of NH_4_^+^ and Al were comparable (some deficit in the NH_4_^+^ content might be due to the fact that not all Na^+^ was exchanged to NH_4_^+^—exchange degree was ca 80%–90%). A similar situation was observed in our previous study for samples treated with NH_3_ solution [20], where comparable concentrations of NH_4_^+^ and Al were also reported (of 510 and 500 cm/µmol, respectively). A good agreement between the amounts of NH_4_^+^ and Al confirmed the hypothesis that all Al take tetrahedral AlO_4_^−^ form in a processed sample and can generate acidity (vide infra). It concerned both zeolitic Al_IVa_ (NMR signal at ca. 60 ppm) and nonzeolitic Al_IVb_ one (NMR signal at ca. 58 ppm).

**Figure 4:**
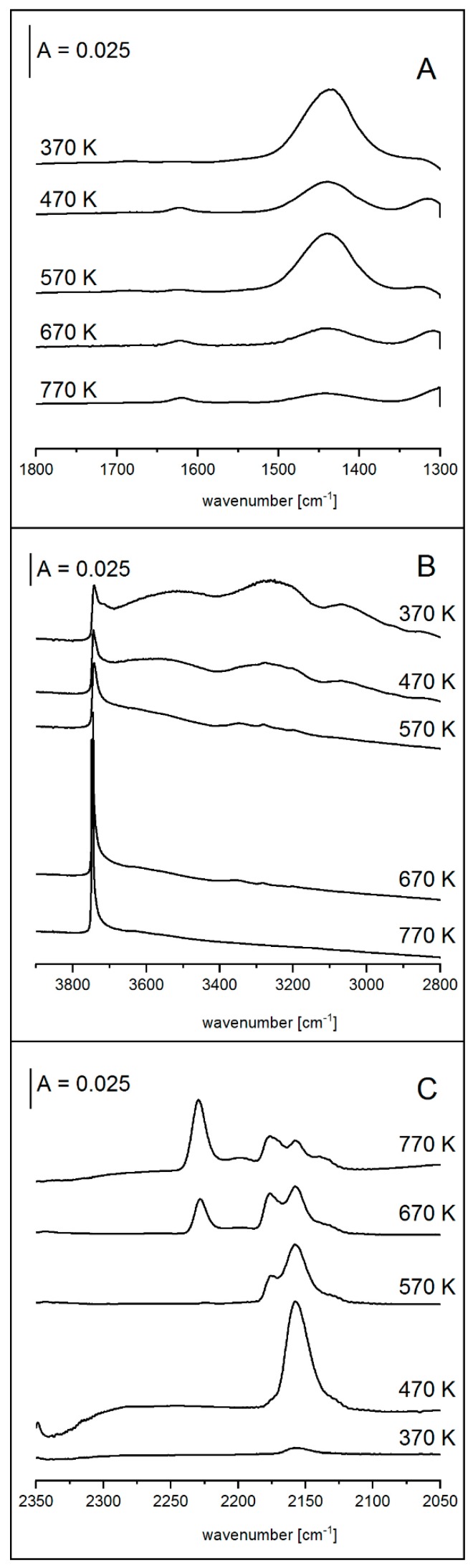
IR spectra of NaOH NH_4_^+^ sample presented in ammonium ions region (**A**), in OH region (**B**), and after CO sorption (**C**), upon heating.

The NH_4_^+^ ions decomposed at 470 K (the 1450 cm^−1^ band diminished). As stated above, the decomposition of NH_4_^+^ ions should produce acidic hydroxyls. Although a part of them dehydroxylate, forming water molecules and Lewis acid sites, some of them might be retained as Brønsted acid sites. Such a hypothesis was further supported by the results of the experiment in which zeolite samples were treated with NaOH and subsequently transformed into ammonium form (by NH_3_ sorption), were heated in vacuum at 370, 470, 570, 670, and 770 K. After each activation step, ammonia sorption was done at 400 K (spectra presented in Figure 4A) and the concentrations of NH_4_^+^ ions were again calculated from the intensities of the band at 1450 cm^−1^ and the extinction coefficients (vide supra). The values of concentrations of NH_4_^+^ ions measured in this experiment are presented in Table 4 where the concentrations of NH_4_^+^ before and after NH_3_ sorption are given. Heating at 470, 570, and 670 K decreased the concentration of NH_4_^+^ ions, however, some of them still restored upon NH_3_ sorption indicating that some protonic sites survived (did not dehydroxylate) and could still restore NH_4_^+^. IR spectra recorded in the OH region (Figure 4B), however, showed no band of acidic hydroxyls, which normally appears around 3600 cm^−1^. A similar situation was observed in amorphous aluminosilicates [35].

The formation of a specific type of acidic hydroxyls produced in the desilicated sample after changing the sodium form to NH_4_^+^ form and ammonium ions decomposition at temperatures higher than 470 K, might be understood when the dealumination mechanism accompanied by the dissolving of Si during desilication is considered. The detailed mechanism of dealumination has already been discussed at a molecular level, by Zhai et al. [2] and these authors proposed that in the final steps of the dealumination process, two intermediates of comparable stability are present in the sample dealuminated by NaOH. One of them corresponds to the [Al(OH)_4_]–Na^+^ complex in solution, weakly interacting with the defected zeolite surface where three Si–O– dangling bonds produced by disrupter of three Si–O–Al linkages upon Al removal, are saturated and neutralized by 3 Na^+^ cations. The other species showed a slightly reorganized structure where the [Al(OH)_4_]–Na complex still bound weakly to a surface dangling oxygen. Comparable energies of these two structures again indicated that extra-framework Al forms produced by dealumination tended to stay close to the surface, with a high probability of it being reinserted into zeolite. Moreover, we might speculate that a further structural reorganization might happen after the exchange of Na^+^ by NH_4_^+^ and next by H^+^ cations upon NH_4_NO_3_ treatment and calcination. [Al(OH)_4_]–H^+^ complex in solution, weakly interacting with the defected zeolite surface (exposing three Si–O–H silanol groups) might undergo condensation with one of surface silanols. This condensation would produce water and a surface fragment like (OH)_3_–Al–(OH)–Si≡. The last fragment resembled acidic bridging hydroxyl but the Al-end was saturated by three OH^−^ groups protruding into the solution and the Si-end originated from a silanol nest. This suggested that a kind of surface acidic bridging hydroxyl might have formed in this specific form but showed modified acidic properties that were different from standard Brønsted acid sites.

As mentioned above, dehydroxylation of the protonic sites produced Lewis acid sites. This was evidenced by the presence of the 1620 cm^−1^ band of NH_3_–L and of the 2230 cm^−1^ band of CO, after NH_3_ and CO sorption on the sample, respectively (Figure 4A,C). The concentration of Lewis acid sites was calculated from the intensity of the 1620 cm^−1^ band (with its extinction coefficient determined in our previous study [36]). Condensation of two acidic hydroxyls in zeolites produced one Lewis site, therefore, the sum B + 2L (where B and L represent concentrations of Brønsted and Lewis sites) should have been constant as the dehydroxylation process proceeded. In our case (Table 4), the B + 2L values were practically independent of activation temperature and remained close to the concentration of Al determined by chemical analysis (compare Table 3 and Table 4). It indicated that Lewis acid sites in desilicated zeolites were formed by dehydroxylation of acidic hydroxyls that were tied with tetrahedral, nonzeolitic Al, and that this dehydroxylation occurred according to the stoichiometry typical for zeolites.

It should be noted here that in “normal” (not desilicated) zeolites, the Si–OH–Al groups are more stable and resistant to dehydroxylation (the calcination below 750 K causes only a very small loss of protonic acidity).

The effect of calcination on the status of Al was also followed by ^27^Al MAS NMR spectroscopy for the samples calcined at 770 K (Figure 2B,D,F, and Table 2). Calcination did not influence the state of aluminum in the parent sample but diminished the contribution of the four-coordinated Al (both Al_IVa_ and Al_IVb_) and produced hexa-coordinated Al forms, mainly Al_VIb_ and Al_VIc_, in desilicated samples. It is possible that these hexa-coordinated Al forms corresponded to the Lewis acid sites formed by the dehydroxylation of protonic sites. Other two types of Al, A_lVa_, and Al_VIa_, were present in the samples before the calcination, and experience showed almost no change after the thermal treatment at 770 K. The reason could be that these types of Al did not take part in a cation-induced coordination change, as was noticed by Altwasser et al. for a certain type of Al_VI_ with CQ = ca. 5 MHz [34]. After the calcination, the Al_IV_ signals returned to their former positions from before the desilication, i.e., 60.8 ppm and 58.6 ppm for Al_IVa_ and Al_IVb_, respectively. For the NaOH desilicated samples, the Al_IVb_ peak got considerably wider, which could be connected to a higher degree of amorphization, and therefore, the existence of various angles between Si–O–Al in amorphous aluminosilicate produced during the calcination.

## 3. Materials and Methods

The parent zeolite Y with Si/Al = 31 (CBV 760) was supplied by Zeolyst (Kansas City, USA). Desilication was carried out by using a 0.2 M NaOH and NaOH/TBAOH mixture containing 10 mol % of TBAOH (total 0.2 M) for 30 min. The desilication temperature was 353 K and the mass ratio of a solution to zeolite was 30. After desilication, the suspension was cooled down in ice-bath, filtered, and washed until neutral pH was obtained. Fourfold Na^+^/NH_4_^+^ ion-exchange with 0.5 M NH_4_NO_3_ was subsequently performed at 60 °C for 1 h. Afterwards, the samples were filtrated again, washed, and dried at room temperature. Finally, the zeolites were calcined in air flow at 790 K for 10 h.

The X-ray powder diffraction (XRD) was recorded with a PANalytical X’Pert PRO MPD diffractometer (PANanalytical, Almelo, Netherlands) with X’Celerator detector type, at room temperature. The measurements were carried out continuously over a 2θ range from 5 to 50° with a 0.0167° and at a time per step of 29.84 s. CuKα radiation (λ = 1.5418 Å) at 40 kV and 30 mA was used. The same amount of samples was placed in the holders prior to data acquisition. Crystallinity was calculated as the sum of integrated area of reflexes at 15.7, 20.5, 27.2, 29.9, and 34.4° [37].

Si, Al, and Na contents were determined by ICP OES spectroscopy on an Optima 2100DV (PerkinElmer, Arcon, OH, USA) instrument. In order to determine the composition of zeolites, 70–80 mg of a zeolite sample was treated with the mixture of 0.3 mL HF and 3 mL of concentrated HCl in a Teflon vessel for 24 h. After the dissolution of zeolite, the liquid was diluted to 50 mL and the Si, Al, and Na amounts were determined by ICP OES spectroscopy. The exchange degree Na/NH_4_ was 80% and 90% for the samples desilicated with NaOH and NaOH/TBAOH, respectively. The accuracy of measurement was ca. 5%–10%.

Prior to IR experiments, self-supported zeolite wafers (diameter of 1 cm, m = 10–20 mg) were evacuated in situ in an IR cell at various temperatures, for 1 h. The spectra were recorded with a NICOLET 7600 (Cambridge, MA, USA) spectrometer with a spectral resolution of 1 cm^−1^. The adsorption of carbon monoxide was performed at 170 K. The concentration of Brønsted and Lewis acid sites was determined quantitatively by IR spectroscopy of the adsorbed ammonia at 400 K.

The ^27^Al solid state Magic-Angle-Spinning Nuclear Magnetic Resonance (MAS NMR) spectra were acquired on a Bruker Avance III 500 MHz WB spectrometer (Bruker BioSpin GMBH, Rheinstetten, Germany), operating at a magnetic field of 11.7 T. Prior to the experiments, the samples were exposed to the vapor of a saturated Mg(NO_3_)_2_ solution at ambient temperature. The ^27^Al investigations were performed on fully hydrated samples, at resonance frequency of 130.33 MHz, using short 0.2 μs single-pulse excitations (π/16), and a repetition time of 0.5 s. A 4-mm zirconia rotor and KEL-F cap was used to spin the sample at 12 kHz. A total of 8,192 transients were acquired for a spectrum. ^27^Al chemical shifts were quoted in parts per million from the external 1 M aqueous Al(NO_3_)_3_ solution. All deconvolutions and CQ values were calculated using the Sola package in Topspin software.

## 4. Conclusions

^27^Al MAS NMR spectra revealed 6 kinds of Al in our parent dealuminated zeolite Y (of Si/Al = 31) and in the samples desilicated using NaOH and NaOH/tetrabutylamine hydroxide (TBAOH). They comprised—two tetrahedral forms, Al_IVa_ (typical of a bulk zeolitic framework) and Al_IVb_ (similar to amorphous aluminosilicates), penta-coordinated Al_V_, and three kinds of hexa-coordinated forms, Al_VIa_, Al_VIb_, and Al_VIc_. The distribution of Al among these sites depended on the stage of zeolite treatment. Desilication removed both Si and Al from zeolite, but while the extracted Si species were prone to self-aggregation and remained in solution, the Al species preferred to be adsorbed onto the zeolite surface. In consequence, the contribution of bulk zeolitic Al_IVa_ decreased upon desilication and the contribution of modified Al_IVb_ forms increased. This is important since the effective enrichment of the concentration of surface Al forms is frequently claimed to be the reason behind a hindering of excessive mesopore formation. Quantitative IR study evidenced that Al extracted from zeolite is then reinserted back as tetrahedral Al and showed an ion-exchange capacity. After the exchange of Na^+^ to NH_4_^+^, decomposition of ammonium ions and the subsequent dehydroxylation of the produced hydroxyls, result in the formation of Lewis acid sites. The quantitative IR studies evidenced that the stoichiometry of dehydroxylation was typical of zeolites—loss of two protonic sites produced one Lewis site. The latter findings seemed to carry the main new message from this study. Finally, calcination of the desilicated zeolites decreased the contribution of both Al_IVa_ and Al_IVb_ and produced mostly hexa-coordinated Al forms, mainly Al_VIb_ and Al_VIc_. It is possible that these hexa-coordinated Al species corresponded to Lewis acid sites formed by dehydroxylation of protonic sites.

## Figures and Tables

**Table 1:** The amounts of Si and Al extracted by the treatment with NaOH and NaOH/TBAOH, and textural properties (volume of micro- and mesopores, mesopore surface (S_meso_), and their diameter (D)).

Sample	Si/Al	% Extracted		Pore Volume [cm^3^/g]		S Meso [cm^2^/g]	D [nm]
		Si	Al	Micro	Meso		
FAU-31	31			0.33	0.20	230	3.0
NaOH; NH_4_^+^ calc. (770 K)	11	79	4.4	0.08	0.52	432	4.3
NaOH/TBAOH; NH_4_^+^ calc.	17	44	2.2	0.21	0.89	460	5.3

**Table 2:** Results of spectral deconvolution and calculated quadrupolar constant (CQ) of the samples under study. δ_iso_ is given in ppm.

Sample	Int_m_^1^	Int_t_^2^	Si/Al ^3^	Al_Iva_^4^		Al_IVb_^5^		Al_V_^6^		Al_VIa_^7^		Al_VIb_^8^		Al_VIc_^9^	
				δ_iso_	%	δ_iso_	%	δ_iso_	%	δ_iso_	%	δ_iso_	%	δ_iso_	%
FAU-31	1	1	31	60.8	45	58.6	18	38.6	7	13.8	8	0.4	18	0.0	5
FAU-31 calc.	0.9	n.d.	n.d.	60.8	47	58.6	16	38.6	8	13.8	9	0.4	14	0.0	6
NaOH/TBAOH; NH_4_^+^	2.8	1.8	17	59.9	31	58.1	57	38.6	4	13.8	8	0.4	0	0.0	0
NaOH/TBAOH; NH_4_^+^ calc.	2.6	1.8	17	60.8	25	58.6	40	38.6	5	13.8	1	0.4	25	0.0	4
NaOH; NH_4_^+^	4.2	2.8	11	60.8	7	58.6	78	38.6	5	13.8	9	0.4	0	0.0	0
NaOH; NH_4_^+^ calc. (770 K)	3.1	n.d.	n.d.	60.8	0	58.6	67	38.6	4	13.8	3	0.4	24	0.0	3

^1^ Int_m_—measured integral, ^2^ Int_t_—anticipated integral calculated from Si/Al ratio, ^3^—ICP reproduced from [25], ^4^—CQ = 1.5 MHz, ^5^—CQ = 3.2 MHz, ^6^—CQ = 5.5 MHz, ^7^—CQ = 5.1 MHz, ^8^—CQ = 4.0 MHz, ^9^—CQ = 1.5 MHz.

**Table 3:** Concentration of Al (from chemical analysis) and of NH_4_^+^ (after ion exchange, from IR spectra).

Sample	µmol/g	
	Al	NH_4_^+^
NaOH NH_4_^+^	1200	1050
NaOH/TBAOH NH_4_^+^	800	700

**Table 4:** Concentrations of NH_4_^+^ before and after NH_3_ sorption in calcined samples (**B**) (equal to concentration of Bronsted sites), concentration of Lewis acid sites (**L**) and sum (**B + 2L**). The concentration of NH_4_^+^ in zeolites activated before NH_3_ sorption is given in parentheses.

Activ. Temp [K]	B	L	B + 2L
370	1050	0	1050
470	(620) 830	120	1080
570	(210) 430	363	1156
670	(0)	400	1030
770	(0)	425	980
